# Cannabinoid 1 Receptor Signaling on Hippocampal GABAergic Neurons Influences Microglial Activity

**DOI:** 10.3389/fnmol.2018.00295

**Published:** 2018-08-28

**Authors:** Frank Ativie, Joanna A. Komorowska, Eva Beins, Önder Albayram, Till Zimmer, Andreas Zimmer, Dario Tejera, Michael Heneka, Andras Bilkei-Gorzo

**Affiliations:** ^1^Institute of Molecular Psychiatry, Medical Faculty, University of Bonn, Bonn, Germany; ^2^Department of Neurodegenerative Diseases & Gerontopsychiatry, Medical Faculty, University of Bonn, Bonn, Germany

**Keywords:** microglia activity, GABAergic neurons, CB1 cannabinoid receptor, LPS stimulation, cytokine expression, morphology and size

## Abstract

Microglia, the resident immune cells of the brain, play important roles in defending the brain against pathogens and supporting neuronal circuit plasticity. Chronic or excessive pro-inflammatory responses of microglia damage neurons, therefore their activity is tightly regulated. Pharmacological and genetic studies revealed that cannabinoid type 1 (CB1) receptor activity influences microglial activity, although microglial CB1 receptor expression is very low and activity-dependent. The CB1 receptor is mainly expressed on neurons in the central nervous system (CNS)—with an especially high level on GABAergic interneurons. Here, we determined whether CB1 signaling on this neuronal cell type plays a role in regulating microglial activity. We compared microglia density, morphology and cytokine expression in wild-type (WT) and GABAergic neuron-specific CB1 knockout mice (GABA/CB1^−/−^) under control conditions (saline-treatment) and after 3 h, 24 h or repeated lipopolysaccharide (LPS)-treatment. Our results revealed that hippocampal microglia from saline-treated GABA/CB1^−/−^ mice resembled those of LPS-treated WT mice: enhanced density and larger cell bodies, while the size and complexity of their processes was reduced. No further reduction in the size or complexity of microglia branching was detected after LPS-treatment in GABA/CB1^−/−^ mice, suggesting that microglia in naïve GABA/CB1^−/−^ mice were already in an activated state. This result was further supported by correlating the level of microglial tumor necrosis factor α (TNFα) with their size. Acute LPS-treatment elicited in both genotypes similar changes in the expression of pro-inflammatory cytokines (TNFα, interleukin-6 (IL-6) and interleukin 1β (IL-1β)). However, TNFα expression was still significantly elevated after repeated LPS-treatment in WT, but not in GABA/CB1^−/−^ mice, indicating a faster development of tolerance to LPS. We also tested the possibility that the altered microglia activity in GABA/CB1^−/−^ mice was due to an altered expression of neuron-glia interaction proteins. Indeed, the level of fractalkine (CX3CL1), a neuronal protein involved in the regulation of microglia, was reduced in hippocampal GABAergic neurons in GABA/CB1^−/−^ mice, suggesting a disturbed neuronal control of microglial activity. Our result suggests that CB1 receptor agonists can modulate microglial activity indirectly, through CB1 receptors on GABAergic neurons. Altogether, we demonstrated that GABAergic neurons, despite their relatively low density in the hippocampus, have a specific role in the regulation of microglial activity and cannabinoid signaling plays an important role in this arrangement.

## Introduction

Microglia the resident immune cells of the central nervous system (CNS), continuously survey their microenvironment (Nimmerjahn et al., 2005) and contribute to the neuronal circuit plasticity by phagocyting apoptotic neurons (Neumann et al., 2009) and by participating in synaptic stripping (Wake et al., 2009). In the presence of a pro-inflammatory stimulus they become activated and are responsible for the active immune defense within the CNS (Schuitemaker et al., 2012; Kaunzner et al., 2012). A long-lasting or excessive inflammatory response is neurotoxic leading to cognitive impairments (Tha et al., 2000; Lynch, 2010), promotion of neurodegeneration (Perry et al., 2010) and brain ageing (Finch, 2010; Villeda et al., 2011). Thus, microglial activity is tightly controlled and fine-tuned in the brain (Wolf et al., 2008). Neurons exert an inhibitory control on the immune activity of microglia (Chavarría and Cárdenas, 2013) through the expression of soluble regulatory factors (Pocock and Kettenmann, 2007) and directly interacting surface proteins (Hoek et al., 2000; Cardona et al., 2006; Bessis et al., 2007).

Pharmacological studies showed a significant effect of cannabinoids on microglial activity. Cannabinoid receptor agonists generally have a neuroprotective, anti-inflammatory effect. *In vitro* studies using cultured microglia showed that activation of the cannabinoid receptors by their endogenous ligand anandamide (Tham et al., 2007) or by synthetic receptor agonists (Romero-Sandoval et al., 2009) inhibited the production of pro-inflammatory mediators and microglia migration. Furthermore, *in vivo* modulation of cannabinoid receptor signaling influenced microglial activity in models of neuroinflammation and neurodegeneration. In the brain of lipopolysaccharide (LPS)-treated (Marchalant et al., 2007) or in the spinal cord of paclitaxel-treated rats (Burgos et al., 2012) the CB1/CB2 receptor agonist WIN-55212-2 reduced the pro-inflammatory activity of microglia cells. In the mouse model of experimental autoimmune encephalomyelitis the synthetic agonist CB52 slowed down Ribeiro et al. (2013), while the cannabinoid receptor antagonist SR141716A accelerated the progression of neuroinflammatory changes (Lou et al., 2018), both in a CB1 receptor-dependent manner. In another study, delta-9-tetrahydrocannabinol (THC) prevented 3,4-Methylendioxy-N-methylamphetamin (MDMA) induced glial activation in wild-type (WT) control mice but not in mice with either genetic or pharmacologic blockade of CB1 receptors (Touriño et al., 2010), further supporting the importance of CB1 receptors in the anti-inflammatory effect of cannabinoid agonists.

The *in vivo* efficacy of CB1 agonists and antagonists is surprising, because in resting/surveying microglia the expression of cannabinoid receptors is very low, often below the detection threshold (Stella, 2010). Microglia upregulates expression of cannabinoid receptors when activated, therefore CB1 ligands could have a direct effect on activated microglia in the late phase of the inflammation. However, the cannabinoid agonist KN38-72717 showed a higher efficacy of in the early phase rather than in the late phase of the neuroinflammatory process (Schmidt et al., 2012). Independent from the activity state, the expression of CB2 receptors significantly exceeds the expression of CB1 receptors (Stella, 2010). Activation of glial CB2 receptors attenuates glial activity (Racz et al., 2008), prevents neurodegeneration (Ullrich et al., 2007) and reduces symptoms of Huntington’s disease (Palazuelos et al., 2009). These data together suggest that *in vivo* the direct effect of cannabinoids on glial activity is mediated primarily by CB2 and not by CB1 receptors. As neurons control microglia cells (Chavarría and Cárdenas, 2013), CB1 receptor agonists and antagonists can influence microglial activity indirectly, by binding neuronal CB1 receptors.

In the brain, the majority of CB1 expressing cells are neurons. In the cortex and in the hippocampus high CB1 receptor expressing cells are GABAergic neurons, whereas glutamatergic principal neurons express CB1 receptors on a lower level (Marsicano and Lutz, 1999). Histological analysis revealed that roughly 90% of cholecystokinin-positive and 10% of calbindin-positive interneurons express CB1 receptors on a high level (Katona et al., 1999). Other GABAergic neuron subtypes like the majority of the parvalbumin-positive interneurons are CB1 negative (Katona et al., 1999).

It is of note that the G-protein coupling of CB1 receptors is several fold more efficient in glutamatergic than in GABAergic neurons, therefore the efficacy of cannabinoids is also higher in this neuronal population (Steindel et al., 2013). Indeed, the importance of CB1 receptors on glutamatergic neurons in neuroprotection shows that deletion of CB1 receptors on glutamatergic but not on GABAergic neurons exacerbates quinolinic acid-induced excitotoxic damage and the progression of striatal neurodegeneration in a mouse model of Huntington’s disease (Chiarlone et al., 2014).

Glial cells not only receive cannabinoid signals but they also express the enzymes involved in the synthesis and degradation of endocannabinoids (Carrier et al., 2004; Walter et al., 2004; Muccioli et al., 2007). They produce cannabinoids such as palmitoylethanolamide (Muccioli and Stella, 2008) and also the endogenous ligands of the cannabinoid receptors anandamide (Walter et al., 2003) and 2-AG (Witting et al., 2004). The 2-AG production is twenty times higher in microglia than in neurons or astrocytes (Walter et al., 2003). The high production of cannabinoids together with the high expression level of CB1 receptors on neurons—especially on GABAergic interneurons—suggests that the cannabinoid system can have a role in microglia-neuron communication (Luongo et al., 2010).

A possible role of neuronal CB1 receptors in the regulation of microglia shows that constitutive CB1 receptor knockouts (Bilkei-Gorzo et al., 2005, 2012) and also GABAergic neuron-specific CB1 knockout mice (Albayram et al., 2011) displayed neuroinflammatory changes in the hippocampus in ageing. An enhanced pro-inflammatory environment is known to contribute to cognitive deficits (Lucin and Wyss-Coray, 2009; Ron-Harel and Schwartz, 2009; Von Bernhardi et al., 2010). Thus, increased pro-inflammatory activity of microglia could be responsible for the reduction of long term potentiation (Monory et al., 2015) and memory deficits (Albayram et al., 2016) in the CB1 knockout lines.

However, it is not yet known how CB1 receptor signaling on GABAergic neurons influences microglial activity in the healthy brain and their activation by a pro-inflammatory stimulus. Therefore, we compared microglia density, size and cytokine expression in WT and GABAergic neuron-specific CB1 knockout mice and compared the dynamics of microglia activation in response to LPS-treatment between the genotypes.

## Materials and Methods

### Animals

The experiments were carried out with 3-month-old male and female B6.cg Cnr1 tm1.2Ltz × Tg(dlx6a- cre)1Mekk mice (Monory et al., 2006). In these mice, conditional deletion of the floxed *Cnr1* gene (encoding for CB1) is mediated by the expression of a cre recombinase in forebrain GABAergic neurons (here called GABA/CB1^−/−^). Cre transgenic mice therefore lack CB1 receptors in GABAergic forebrain neurons. *Cnr1* floxed littermates were used as WT controls.

Mice were group housed under reversed light/dark cycle. Food and water were provided *ad libitum*. Care of the animals and conduction of the experiments followed guidelines of European Communities Directive 86/609/EEC and the German Animal Protection Law regulating animal research and were approved by the LANUV NRW, Germany (Nr. 84-02.04.2014.A422).

### *In vivo* LPS Injections and Preparation of the Brain

Mice received acute or repeated (once daily for four consecutive days) intraperitoneal injections of 0.8 mg/kg LPS (Sigma) or saline (control groups). Three or 24 h after the acute treatment or 24 h after the last injection the mice were sacrificed. For that, mice were deeply anesthetized with isoflurane and transcardially perfused with ice cold PBS. Brains were halved and briefly washed in PBS. For the quantitative real-time PCR one hemisphere was immersed in 1ml of TRIzol in Magna Lyser tubes and snap frozen. For immunoblotting, hippocampi of control mice were isolated and shock frozen on dry ice. For histological analysis the half brains were post-fixed in 4% PFA in PBS (pH = 6.9; Sigma-Aldrich) for 3–4 h at 4°C, under shaking (Notter et al., 2014). Hemispheres were then briefly washed with PBS and cryoprotected by incubation in 20% sucrose solution for up to 2 days. After that time, the excess of the sucrose solution was removed and hemispheres were deep frozen in dry-ice cooled isopentane. Samples were stored at −80°C until further processing.

### Quantitative Real-Time PCR

The half brains (excluding the olfactory bulb) were lysed in TRIzol (Life Technologies), and total RNA was extracted according to the manufacturer’s protocol. The quality of the RNA was assessed by measuring the ratio of the absorbance at 260 nm and 280 nm using a Nanodrop 2,000 Spectrometer (Thermo Scientific). Probes with a 260/280 ratio less than 1.9 were rejected. cDNAs were synthesized using the SuperScript First-Strand Synthesis System for RT-PCR Kit (Invitrogen Corp., Carlsbad, CA, USA) with random hexamer primers. Total RNA (0.6 μg) was used as starting material for cDNA synthesis. Differences in mRNA expression were determined in triplicate by custom TaqMan^®^ Gene Expression Assays (Applied Biosystems, Darmstadt, Germany; interleukin-6 (IL-6): Mm00446190_m1; interleukin 1β (IL-1β): Mm00434228_m1; tumor necrosis factor (TNF): Mm00443258_m1. The glyceraldehyde-3-phosphate dehydrogenase (GAPDH): Mm01334042_m1 was used as an endogenous reference gene to standardize the amount of target cDNA. Typically, a reaction mixture consisted of 1× TaqMan^®^ Gene Expression Master Mix (Applied Biosystems, Darmstadt, Germany), 2 μl cDNA and 1× Custom TaqMan^®^ Gene Expression Assay. Samples were processed in a 7500 Real-Time PCR Detection System (Applied Biosystems, Darmstadt, Germany) with the following cycling parameters: 95°C for 10 min (hot start), 40 cycles at 95°C for 15 s (melting) and 60°C for 1 min (annealing and extension). Analysis was performed using the 7500 Sequence Detection Software version 2.2.2 (Applied Biosystems, Darmstadt, Germany) and data were obtained as function of threshold cycle (Ct).

Relative quantitative gene expression was calculated with the 2^−ddCt^ method. Briefly: dCt was calculated for each assayed sample by subtracting Ct of the housekeeping gene from the Ct of the gene of interest. Mean dCt values of the saline-treated WT mice were chosen as reference sample and subtracted from dCt of the other groups (ddCt). Experimental mRNA abundance relative to control mRNA abundance was finally calculated. The animal numbers used for this analysis were as follows: single LPS injection, killed 3 h later: seven in each group. Single LPS injection, killed 24 h later: eight. Repeated LPS injection, killed 24 h after the last injection: six. We had to exclude one probe in the WT saline-treated groups due to a technical error.

### Immunohistochemistry

Frozen fixed hemispheres were cut with a cryostat (Microm HM500) into 18 μm slices and stored at −20°C. For the staining, the slices were first permeabilized with 0.5% Triton X-100 in PBS and unspecific binding sites were blocked with 3% BSA in PBS. Slices were incubated with the primary antibody for 48 h at 4°C and after washing with the secondary antibody overnight. Primary antibodies: rabbit anti-Iba1 (1:2,000, Wako, 019-19741), mouse anti-TNFα (1:100, Abcam, ab1793), mouse anti-GAD67 (1:1,000, Abcam, ab26116) and rabbit anti CX3CL1 (1:500, Abcam, ab25088). Secondary antibodies: donkey anti-rabbit conjugated with Cy3 (1:2,000; Invitrogen; A1-520), donkey anti-rabbit conjugated with AF488 for the Iba1/TNFα double staining experiment (1:1,000; Invitrogen, A21206), donkey anti-rabbit conjugated with AF594 for the GAD67/CX3CL1 double staining (1:1,000, Invitrogen, A21207), donkey anti-mouse AF647 conjugated for the Iba1/TNFα double staining (1:1,000; Invitrogen; A31571), donkey anti-mouse AF488 conjugated for the GAD67/CX3CL1 double staining (1:1,000; Invitrogen; A21202). Finally, slices were washed, embedded with DAPI containing medium (DAPI Fluormount-G R, Southern Biotech) and covered. To test microglia densities, we analyzed slices from six mice in the single injected groups, six WT vehicle-injected and GABA/CB1^−/−^ LPS injected, four WT LPS-injected and five GABA/CB1^−/−^ single injected mice. For the analysis of microglia morphology, microglial TNF alpha expression and GAD67 – CX3CL1 colocalization we used three mice for each group.

### Imaging and Image Analysis

For the analysis of microglia densities within the hippocampus, images were acquired using a Zeiss Axio Imager two epifluorescent microscope with 20× objective. To determinate microglia density, the number of Iba1-positive (Iba1+) cells within the hippocampus was counted and divided by the area of the hippocampus. The average density was calculated from six images per mouse, 4–6 mice were analyzed per group.

For the analysis of microglia morphology and TNFα immunoreactivity, as well as GAD67/CX3CL1 double stainings, images were acquired using a Leica TCS SP8 confocal microscope with 63× objective. Z-stacks were taken with a step size of 0.5 μm.

To determine microglia morphology, microglia cells were automatically reconstructed in a three-dimensional way after imaging acquisition. In order to this, a python-based software was developed. Reconstruction were visually checked using the ImageJ plugin “simple neurite tracer (ImageJ V2.0).” Each reconstructed cell was individually extracted and the number and length of branches, bifurcations and branch order were quantified using the open source software l-measure (Scorcioni et al., 2008). 20–25 randomly selected microglia from three different slices were analyzed per mouse, three mice per group, giving a total of 60–72 microglia per group.

The intensity of TNFα immunoreactivity in 20–25 microglia per mouse was measured. For this analysis high magnification histological pictures of microglia cells were taken in the hippocampus of 3–3 wild-type and GABA/CB1^−/−^ saline treated mice, thus altogether 60–72 microglia cells were evaluated using the Fiji software (ImageJ V2.0).

CX3CL1 immunoreactivity within hippocampal GAD67+ neurons was measured in WT and GABA/CB1^−/−^ saline treated mice (*n* = 3 per group, altogether 75 neurons), using the Fiji software (ImageJ V2.0).

### Determination of Neuron-Glia Interaction Protein Levels Using Western Blotting

Frozen hippocampi of untreated WT and GABA/CB1^−/−^ mice were lysed in 1% SDS buffer (Sigma-Aldrich, Munich, Germany) containing protease inhibitor (Complete Mini, Roche), sonicated and clarified by centrifugation (13,000 rpm for 10 min). Protein concentrations were determined using BCA Protein Assay Kit (Pierce). Equal amounts of protein were run on NuPAGE Bis-Tris 4%–12% gradient gels (Invitrogen, Carlsbad, CA, USA). In preliminary experiments we determined the range where the relation between the protein concentration and signal intensity is linear for each antibody. The proteins were subsequently blotted onto PVDF-membranes using the iBlot Dry Blotting System (Invitrogen, Carlsbad, CA, USA). The blots were incubated with primary antibodies to CD200 (bs-6030R; 1:1,000; Bioss Antibodies), CD200R (1:1,000; ab34097; Abcam), CX3CL1 (1:1,000; ab25088; Abcam), CX3CR1 (1:1,000; C8354; Sigma-Aldrich), Sirpa (1:1,000; ab53721; Abcam), CD47 (1:1,000; sc25773; Santa Cruz) and with an antibody against GAPDH (1:12,000; ab9484; Abcam). The blots were then incubated with peroxidase-conjugated secondary antibodies, followed by the ECL substrate (Pierce). Images were created using the ChemiDoc Imaging System (Bio-Rad Laboratories) and the quantification was performed using the ImageLab software (Bio-Rad Laboratories). Signal intensities were normalized to GAPDH.

### Statistical Analysis

Statistical analysis was performed and visualized using GraphPad Prism 6.0. For the gene expression analysis, to test hippocampal microglia densities, sizes and morphology two-way ANOVA (main factors: genotype, treatment) followed by Bonferroni test was used. Additionally, we analyzed the effect of sex on microglia activation in mice sacrificed 24 h after a single LPS injection using three-way ANOVA main factors: genotype, treatment, sex). For the analysis of correlation between microglia size and TNFα content Pearson correlation analysis was used. To compare the microglial TNFα levels, the level of CX3CL1 in GABAergic neurons between the genotypes Mann-Whitney test was used, for the analysis of Western blot data the Student *t*-test. *P-value < 0.05 was* considered as significant.

## Results

### Effect of Intraperitoneal LPS Injection

To compare the dynamics of the inflammatory reaction between WT and GABA/CB1^−/−^ mice, we injected with 0.8 mg/kg LPS intraperitoneally and evaluated the gene expression level of pro-inflammatory cytokines in the brain three and 24 h after a single injection and 24 h after a repeated daily injection of LPS for 4 days.

### Expression of Inflammatory Cytokines

**Figure 1:**
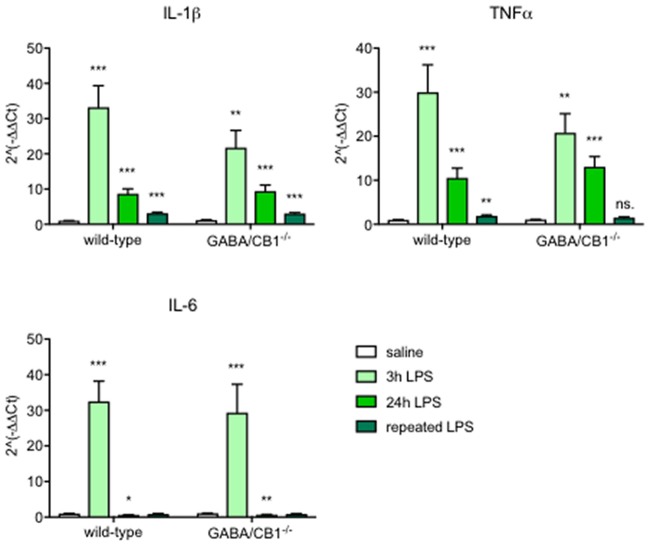
Expression of inflammatory cytokines in the brain of wild-type (WT) and GABA/CB1^−/−^ mice; control groups are pulled together and shown as white bars (*n* = 21), 3 h after an intraperitoneal lipopolysaccharide (LPS)-treatment (*n* = 7) shown as light green bars, 24 h after an intraperitoneal LPS-treatment (*n* = 7–8) shown as intensive green bars and 24 h after the fourth daily intraperitoneal LPS injection (*n* = 6) shown as dark green bars. ns. stands for not significant; *p < 0.05; **p < 0.01; ***p < 0.001 difference detected by Bonferroni’s *t*-test between LPS and saline-treated mice from the respective control group.

The expression of the proinflammatory cytokines IL-6, IL-1β and TNF α, each showed a strong increase 3 h after the LPS injection in both genotypes (Figure 1, Table 1A). When we tested the expression levels in the later phase of the reaction, 24 h after the LPS treatment, we found again a similar change in LPS-treated mice, independent from the genotype: reduction in the IL-6 and elevation in the IL-1 β and TNFα mRNA levels (Figure 1, Table 1B). Sex of the mice also did not influence the cytokine expression (Table 1B). Twenty-four hours after the repeated, daily injection of LPS the IL-6 levels did not differ between the LPS-treated and vehicle-treated controls. The IL-1 β and TNFα mRNA levels were elevated in the LPS-treated WT mice, whereas in GABA/CB1^−/−^ mice the expression of TNF α was similar in vehicle- and LPS-treated mice (Figure 1, Table 1C).

**Table 1:** Detailed results of ANOVA analysis of the expression of inflammatory cytokines (A) 3 h; (B) 24 h of a single lipopolysaccharide (LPS) injection and (C) 24 h after repeated LPS injections.

IL-1β	IL-6	TNFα
**A: Expression of inflammatory cytokines 3 h after the LPS injection**		
Treatment effect: *F*_(1,24)_ = 45.30; *p* < 0.001	Treatment effect: *F*_(1,24)_ = 37.70; *p* < 0.001	Treatment effect: *F*_(1,24)_ = 40.86; *p* < 0.001
Genotype effect: *F*_(1,24)_ = 2.014; *p* > 0.05	Genotype effect: *F*_(1,24)_ = 0.099; *p* > 0.05	Genotype effect: *F*_(1,24)_ = 1.392; *p* > 0.05
Treatment × genotype interaction: *F*_(1,24)_ = 2.314; *p* > 0.05	Treatment × genotype interaction: *F*_(1,24)_ = 0.111; *p* > 0.05	Treatment × genotype interaction: *F*_(1,24)_ = 1.547; *p* > 0.05
**B: Expression of inflammatory cytokines 24 h after the LPS injection**		
Treatment effect: *F*_(1,23)_ = 45.31; *p* < 0.001	Treatment effect: *F*_(1,23)_ = 11.74; *p* < 0.01	Treatment effect: *F*_(1,23)_ = 39.46; *p* < 0.001
Genotype effect: *F*_(1,23)_ = 0.117; *p* > 0.05	Genotype effect: *F*_(1,23)_ = 0.457; *p* > 0.05	Genotype effect: *F*_(1,23)_ = 0.451; *p* > 0.05
Sex effect: *F*_(1,23)_ = 0.270; *p* > 0.05	Sex effect: *F*_(1,23)_ = 0.040; *p* > 0.05	Sex effect: *F*_(1,23)_ = 1.352; *p* > 0.05
Treatment × genotype interaction: *F*_(1,23)_ = 0.088; *p* > 0.05	Treatment × genotype interaction: *F*_(1,23)_ = 0.292; *p* > 0.05	Treatment × genotype interaction: *F*_(1,23)_ = 0.609; *p* > 0.05
Treatment × sex interaction: *F*_(1,23)_ = 0.293; *p* > 0.05	Treatment × sex interaction: *F*_(1,23)_ = 0.097; *p* > 0.05	Treatment × sex interaction: *F*_(1,23)_ = 1.063; *p* > 0.05
Genotype × sex interaction: *F*_(1,23)_ = 1.193; *p* > 0.05	Genotype × sex interaction: *F*_(1,23)_ = 0.400; *p* > 0.05	Genotype × sex interaction: *F*_(1,23)_ = 0.324; *p* > 0.05
Treatment × genotype × sex interaction: *F*_(1,23)_ = 1.293; *p* > 0.05	Treatment × genotype × sex interaction: *F*_(1,23)_ = 0.001; *p* > 0.05	Treatment × genotype × sex interaction: *F*_(1,23)_ = 0.422; *p* > 0.05
**C: Expression of inflammatory cytokines after repeated LPS injections**		
Treatment effect: *F*_(1,20)_ = 96.39; *p* < 0.001	Treatment effect: *F*_(1,20)_ = 3.876; *p* > 0.05	Treatment effect: *F*_(1,20)_ = 12.83; *p* < 0.01
Genotype effect: *F*_(1,20)_ = 0.014; *p* > 0.05	Genotype effect: *F*_(1,20)_ = 0.319; *p* > 0.05	Genotype effect: *F*_(1,20)_ = 0.716; *p* > 0.05
Treatment × genotype interaction: *F*_(1,20)_ = 0.324; *p* > 0.05	Treatment × genotype interaction: *F*_(1,20)_ = 0.501; *p* > 0.05	Treatment × genotype interaction: *F*_(1,20)_ = 2.627; *p* > 0.05

### Microglia Density

**Figure 2:**
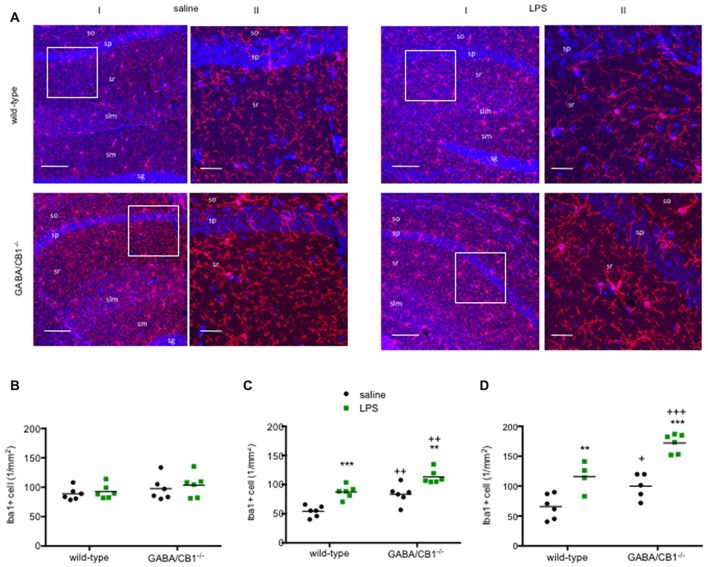
**(A)** Representative microphotograph of Iba1 immunostaining (red) and DAPI fluorescence showing cell nuclei (blue) in the hippocampus of WT and GABA/CB1^−/−^ mice 24 h after four times daily intraperitoneal LPS injections. **(I)** Low magnification image, the scale bar represents 100 μm. White quadrant shows the region presented as high magnification image. **(II)** High magnification image, the scale bar represents 25 μm. so—stratum oriens; sp—stratum pyramidale; sr—stratum radiatum; slm—stratum lacunosum moleculare; sg—stratum granulosum. Density of Iba1-positive (Iba1+) microglia cells in the hippocampus of WT and GABA/CB1^−/−^ mice **(B)** Three hours after an intraperitoneal LPS-treatment. **(C)** Twenty-four hours after an intraperitoneal LPS-treatment. **(D)** Twenty-four hours after four times daily intraperitoneal LPS injections. **p < 0.01; ***p < 0.001 difference between saline and LPS-treated mice using Bonferroni’s *t*-test. ^++^*p* < 0.01; ^+++^*p* < 0.001 difference between WT and GABA/CB1^−/−^ mice with the same treatment using Bonferroni’s *t*-test. ^+^*p* < 0.05.

As a further readout to assess microglial reactivity we compared the density of Iba1+ cells in the hippocampus between saline and LPS-treated mice from both genotypes. As shown on the representative images (Figure 2A) the Iba1+ microglia cells had the highest density in the stratum lacunosum moleculare layer in each group. Microglia densities did not differ between WT and GABA/CB1^−/−^ mice (genotype effect: *F*_(1,20)_ = 2.140; *p* > 0.05) or between saline and LPS-treated mice (treatment effect: *F*_(1,20)_ = 0.528; *p* > 0.05) in both genotypes (interaction: *F*_(1,20)_ = 0.026; *p* > 0.05) 3 h after the LPS-treatment (Figure 2B). Twenty-four hours after the LPS injection, however, we found a significant increase in microglia densities in the LPS-treated group (treatment effect: *F*_(1,20)_ = 37.78; *p* < 0.001), similarly in WT and GABA/CB1^−/−^ mice (treatment × genotype interaction: *F*_(1,20)_ = 0.115; *p* > 0.05; Figure 2C). The effect of LPS was more pronounced in males than in females (treatment × sex: *F*_(1,16)_ = 6.425; *p* < 0.05), similarly in both genotypes (treatment × sex × genotype: *F*_(1,16)_ = 0.031; *p* > 0.05). The density values were higher in the conditional knockouts than in their WT littermates (genotype effect: *F*_(1,20)_ = 29.10; *p* < 0.001) in both sexes (genotype × sex: *F*_(1,16)_ = 0.020; *p* > 0.05). We found a similar picture after repeated LPS-injections as 24 h after the single injection (Figure 2D): significantly increased microglia in the LPS-treated mice (treatment effect: *F*_(1,17)_ = 46.75; *p* < 0.001) in both genotypes (interaction: *F*_(1,17)_ = 1.506; *p* > 0.05) and significantly bigger microglia in GABA/CB1^−/−^ mice (genotype effect: *F*_(1,17)_ = 25.34; *p* < 0.01). We also wanted to know whether microglia densities differ between the genotypes in control, saline-treated mice, but *post hoc* analysis of the data did not give a clear answer. Thus, we re-analyzed density data of the saline-treated mice from all three experiments using two-way ANOVA with genotype and experiment as main factors. This analysis showed that microglia densities are higher in control GABA/CB1^−/−^ than in WT mice (genotype: *F*_(1,29)_ = 17.63; *p* < 0.001; genotype × experiment interaction: *F*_(2,29)_ = 1.862; *p* > 0.05).

### Microglia Morphology

**Figure 3:**
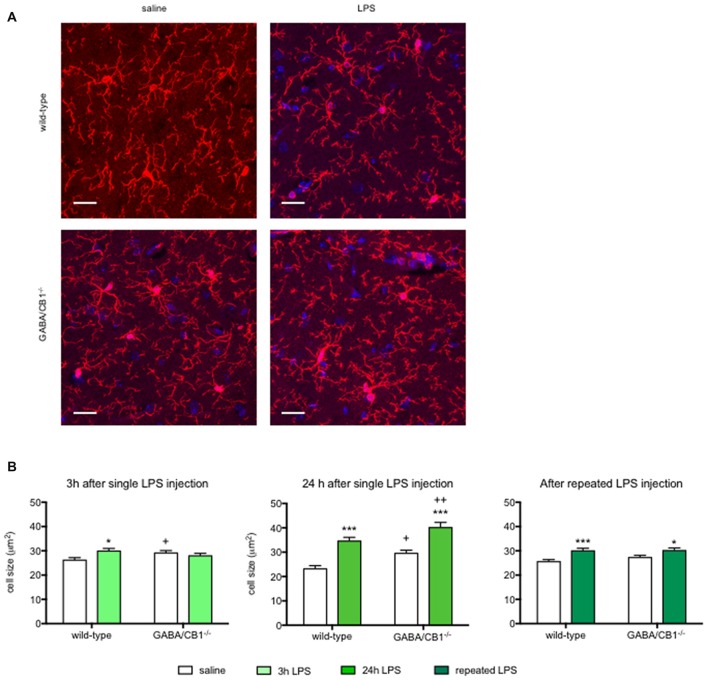
**(A)** Representative microphotograph of Iba1 immunostaining (red) and DAPI fluorescence showing cell nuclei (blue) in the hippocampus of WT and GABA/CB1^−/−^ mice 24 h after four times daily intraperitoneal LPS injections. Scale bar represents 20 μm. **(B)** Size of Iba1+ microglia cells in the hippocampus of WT and GABA/CB1^−/−^ mice 3 h after an intraperitoneal LPS-treatment (*n* = 143–150); 24 h after an intraperitoneal LPS-treatment (*n* = 136–145) and 24 h after four times daily intraperitoneal LPS injections (*n* = 138–150). *p < 0.05; ***p < 0.001 difference between saline and LPS-treated mice using Bonferroni’s *t*-test. ^+^*p* < 0.05; ^++^*p* < 0.01 difference between WT and GABA/CB1^−/−^ mice with the same treatment using Bonferroni’s *t*-test.

Activated microglia have an increased cell body as well as decreased size and complexity of branches (Figure 3A), therefore to further analyze the effect of CB1 receptor signaling in GABAergic neurons on microglial activity state, we compared the microglia sizes, parameters of branch size (number and length of branches) and parameters of branch complexity (number of bifurcations and branch order) in the hippocampus of WT and GABA/CB1^−/−^ mice with and without LPS treatment.

Analysis of microglia sizes (Figure 3B) showed that 3 h after the LPS injection there was no significant main effect for genotype or treatment (genotype effect: *F*_(1,586)_ = 0.393; *p* > 0.05; treatment effect: *F*_(1,586)_ = 2.745; *p* > 0.05), but we found a significant interaction between genotype and treatment (interaction: *F*_(1,586)_ = 9.835; *p* < 0.01). *Post hoc* analysis revealed that the microglia sizes increased after LPS-treatment only in WT mice. It is of note that microglia in control GABA/CB1^−/−^ mice were also somewhat larger than in control WT mice. When we assessed microglia size in mice 24 h after the LPS treatment, we found significant genotype (*F*_(1,553)_ = 16.51; *p* < 0.001) and treatment effects (*F*_(1,553)_ = 65.07; *p* < 0.001), but no interaction (*F*_(1,553)_ = 0.067; *p* > 0.05). Testing the effect of sex on LPS-induced increase in microglia size revealed a rather complex interaction: LPS had a higher effect in females than in males (genotype × sex: *F*_(1,549)_ = 23.30; *p* < 0.001), because microglia sizes were lower in LPS-treated GABA/CB1^−/−^ males than in females (genotype × treatment × sex: *F*_(1,549)_ = 19.21; *p* < 0.001). Lastly, repeated injection of LPS led also to increased microglia sizes (*F*_(1,576)_ = 24.10; *p* < 0.001), which was not influenced by the genotype (interaction: *F*_(1,576)_ = 1.104; *p* > 0.05). There was no genotype effect in this group (*F*_(1,576)_ = 1.468; *p* > 0.05). Similarly, as by the analysis of microglia densities there was a significant difference between the saline-treated WT and GABA/CB1^−/−^ mice in two from the three studies. Thus, to learn whether microglia sizes differ between the genotypes without LPS-treatment we analyzed again our data from the saline-treated groups using two-way ANOVA with genotype and experiment as main effects. This analysis revealed that microglia are bigger in control GABA/CB1^−/−^ than in WT mice (genotype: *F*_(1,863)_ = 24.96; *p* < 0.001) independent from the experiment (genotype × experiment interaction: *F*_(2,863)_ = 2.080; *p* > 0.05).

**Figure 4:**
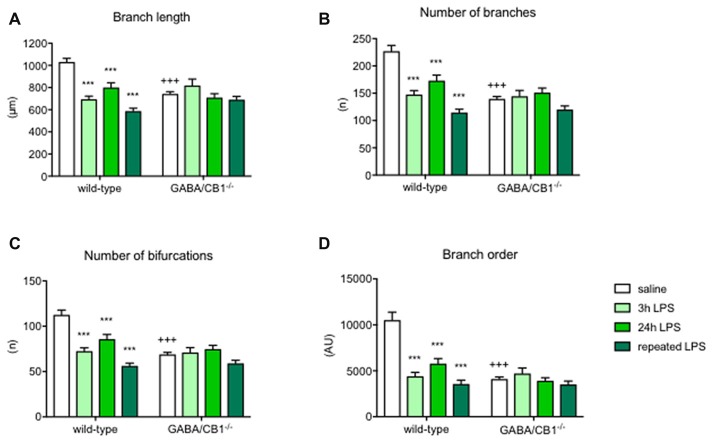
Microglia branch morphology in the hippocampus of WT and GABA/CB1^−/−^ mice^.^ Data of the control groups (saline-treated) are pulled together separately to the genotypes and shown as white bars. Light green bars represent data received 3 h after the intraperitoneal LPS-treatment, Intensive green bars represent data received 24 h after the intraperitoneal LPS-treatment and dark green bars represent data received 24 h after the fourth daily intraperitoneal LPS injection. Extent of branching is shown as **(A)** Branch length **(B)** Number of branches. The complexity of microglia branching is shown as **(C)** Number of bifurcations and **(D)** Branch order. *n* = 48–58. ***p < 0.001 difference detected by Bonferroni’s *t*-test between LPS and saline-treated mice from a respected control group; ^+++^*p* < 0.001 difference between WT and GABA/CB1^−/−^ mice with the same treatment, both using two-way ANOVA followed by Bonferroni’s *t*-test.

Detailed analysis of microglia branch morphology in mice 3 h after a single injection of LPS or vehicle revealed that the total length of branches was less in GABA/CB1^−/−^ mice (genotype: *F*_(1,209)_ = 5.178; *p* < 0.05). LPS-treatment reduced branch length (treatment:*F*_(1,209)_ = 15.17; *p* < 0.001), but this reduction was significant only in the WT (−36.6%) but not in the GABA/CB1^−/−^ mice (genotype × treatment interaction: *F*_(1,209)_ = 25.14; *p* < 0.001; Figure 4A). Testing the number of branches our analysis gave a similar result: less branches (genotype: *F*_(1,209)_ = 20.85; *p* < 0.001) and reduced reactivity to LPS in the GABA/CB1^−/−^ mice (genotype × treatment interaction: *F*_(1,209)_ = 17.58; *p* < 0.001; 38%, significant reduction in WT mice and −2.2% not significant change in the knockouts; Figure 4B). For this parameter the treatment effect was not significant (*F*_(1,209)_ = 3.735; *p* = 0.0546). The complexity of branching as a number of bifurcation differed between the genotypes (*F*_(1,209)_ = 20.57; *p* < 0.001). We did not find a significant treatment effect (*F*_(1,209)_ = 3.533; *p* = 0.0616) but a significant genotype × treatment interaction (*F*_(1,209)_ = 17.64; *p* < 0.001) because LPS-induced a significant reduction in the bifurcation in WT (−28.8%) but not in the knockout mice (+16.4%; Figure 4C). Microglia branch order was influenced by the genotype (*F*_(1,209)_ = 11.62; *p* < 0.001) but not by the treatment (*F*_(1,209)_ = 1.616; *p* = 0.205). The interaction between genotype and treatment was significant (*F*_(1,209)_ = 15.74; *p* < 0.001) because of the different reactivity of microglia in WT (−38.5%) and GABA/CB1^−/−^ mice (+6.8%; Figure 4D).

The differences between the groups were even more pronounced 24 h after the LPS injection. Branch length was influenced by the genotype (*F*_(1,224)_ = 31.57; *p* < 0.001), treatment (*F*_(1,224)_ = 21.43; *p* < 0.001) and there was an interaction between the main factors (*F*_(1,224)_ = 14.47; *p* < 0.001) because microglia in GABA/CB1^−/−^ mice were less reactive to LPS-treatment (−23.1% in WTs and −4.5% in knockouts; Figure 4A). The other marker of branch size, number of branches showed a similar change: genotype effect: (*F*_(1,224)_ = 23.08; *p* < 0.001), treatment effect (*F*_(1,224)_ = 17.48; *p* < 0.001) and a genotype × treatment interaction (*F*_(1,224)_ = 10.49; *p* < 0.01) because of a reduced reactivity of microglia in GABA/CB1^−/−^ mice (−37.4% in WTs and −8.0% in knockouts; Figure 4B). Complexity of branching shown as number of bifurcations was lower in GABA/CB1^−/−^ mice (*F*_(1,224)_ = 23.39; *p* < 0.001) and reduced after LPS treatment (*F*_(1,224)_ = 17.69; *p* < 0.001), but differently between the genotypes (genotype × treatment interaction: *F*_(1,224)_ = 10.53; *p* < 0.01). The reason of the interaction was a reduced reactivity of microglia to LPS in the hippocampi of GABA/CB1^−/−^ mice (WTs: −37.6%; knockouts: −8.2%; Figure 4C). The analysis of the other parameter of branch complexity, branch order gave very similar results: genotype effect: (*F*_(1,224)_ = 31.57; *p* < 0.001), treatment effect (*F*_(1,224)_ = 21.43; *p* < 0.001) and a genotype × treatment interaction (*F*_(1,224)_ = 14.47; *p* < 0.001) due to a reduced reactivity of microglia in GABA/CB1^−/−^ mice (−60.0% in WTs and −17.8% in knockouts; Figure 4D). All these morphological changes were more pronounced in males than in females (treatment × sex interaction—branch length: *F*_(1,177)_ = 28.39; *p* < 0.001; number of branches: *F*_(1,177)_ = 28.69; *p* < 0.001; number of bifurcations: *F*_(1,177)_ = 28.22; *p* < 0.001; branch order: *F*_(1,177)_ = 15.89; *p* < 0.001).

The strong difference in microglia morphology between the genotypes, treatment groups and reactivity of genotypes to LPS was present also after repeated treatments in most of the parameters. Branch length was reduced after the repeated LPS injections (*F*_(1,171)_ = 20.58; *p* < 0.001). We did not find a significant genotype effect (*F*_(1,171)_ = 1.409; *p* < 0.237) but again microglia in GABA/CB1^−/−^ mice showed a reduced reactivity to LPS (genotype × treatment interaction: *F*_(1,171)_ = 16.42; *p* < 0.001; change in WT mice −34.5%, change in knockouts: −2.5%; Figure 4A). The number of branches was influenced by the genotype (*F*_(1,171)_ = 10.63; *p* < 0.01), by the treatment (*F*_(1,171)_ = 23.55; *p* < 0.001) and by an interaction between genotype and treatment (*F*_(1,171)_ = 15.56; *p* < 0.001). The reduction in microglial branch number due to the repeated LPS injections was more intensive in the WT (−37.8%) than in knockout mice (−5.7%; Figure 4B). The number of bifurcations was less in the microglia in GABA/CB1^−/−^ mice (*F*_(1,171)_ = 10.64; *p* < 0.01) and it was reduced after the LPS treatments (*F*_(1,171)_ = 25.08; *p* < 0.001). The reduction differed between the genotypes (genotype × treatment interaction: *F*_(1,171)_ = 15.83; *p* < 0.001) because it was more intensive in WT (−38.6%) than in knockout mice (−7.6%; Figure 4C). The analysis of branch order, the alternative parameter of microglia branch complexity, gave similar results: genotype effect: *F*_(1,171)_ = 21.11; *p* < 0.001), treatment effect: *F*_(1,171)_ = 35.92; *p* < 0.001, genotype × treatment interaction *F*_(1,171)_ = 20.43; *p* < 0.001. Here again, the reduction of branch order after LPS treatment was significant in the microglia of WT mice (−61.2%) but not in the microglia of GABA/CB1^−/−^ mice (−18.3%; Figure 4D).

### Microglial TNFα Levels

**Figure 5:**
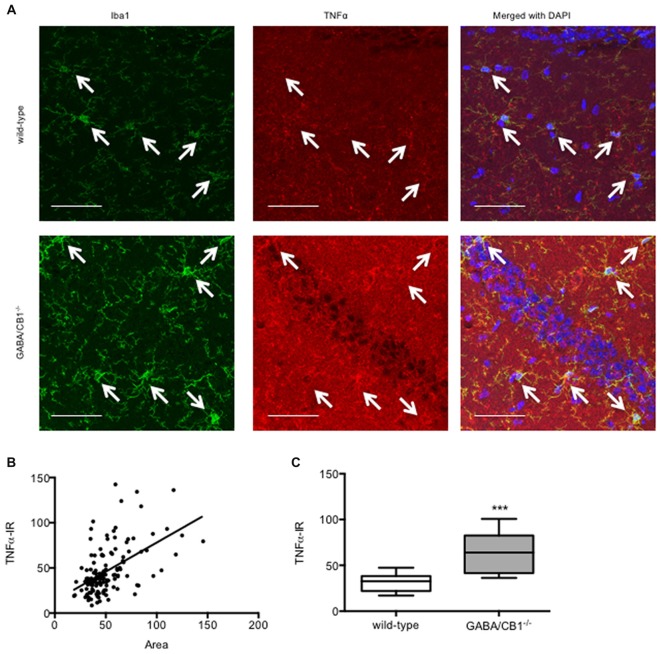
**(A)** Representative microphotograph of confocal images of Iba1 (green) and TNFα (red) colocalization. Scale bar represents 50 μm. White arrows point to Iba1+ microglia. **(B)** Correlation between microglial size and TNFα immunoreactivity. Microglia (*n* = 132) in the hippocampus of saline-treated WT and GABA/CB1^−/−^ mice were analyzed. **(C)** TNFα immunoreactivity of microglia cells in the hippocampus of saline-treated WT and GABA/CB1^−/−^ mice (*n* = 60–72). ***p < 0.001 using Mann-Whitney *U*-test.

Enhanced microglia density and increased cell body are interpreted as signs of microglial activation. Thus, we asked whether the microglial level of the pro-inflammatory cytokine TNFα correlates with the size of the microglia using saline-treated mice from both genotypes. As shown in Figure 5A the TNFα immunoreactivity was higher in the microglia cells than in their surrounding cells or in their microenvironment. Importantly, there was a significant correlation between size and TNFα immunoreactivity of the microglia (*r* = 0.5209, *p* < 0.001; Figure 5B) suggesting that higher cell bodies were associated with enhanced pro-inflammatory activity. In good correlation with our previous finding that microglia in GABA/CB1^−/−^ mice had increased size, the microglial TNFα levels were also higher in GABA/CB1^−/−^ mice than in their WT littermates (Mann-Whitney *U* = 493; *p* < 0.001; Figure 5C).

### Expression of Neuron-Glia Interaction Proteins

**Figure 6:**
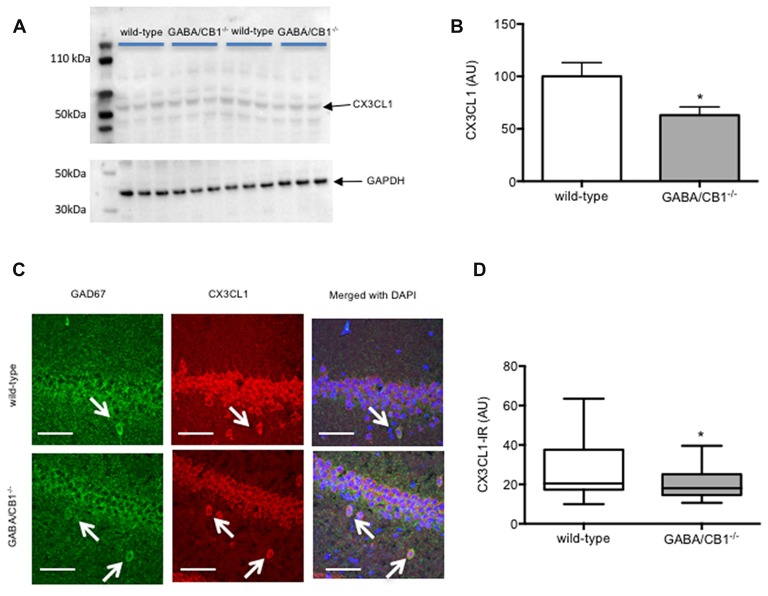
**(A)** Representative images of CX3CL1 and glyceraldehyde-3-phosphate dehydrogenase (GAPDH) immunoblots of hippocampal samples from control WT and GABA/CB1^−/−^ mice. **(B)** Western blot analysis of hippocampal CX3CL1 levels using control WT and GABA/CB1^−/−^ mice. *p < 0.05 using Student’s *t*-test; *n* = 9. **(C)** Representative microphotograph of confocal images of GAD67 (green) and CX3CL1 (red) colocalization. Scale bar represents 50 μm. White arrows point to GAD67-positive GABAergic neurons. **(D)** CX3CL1 immunoreactivity in GAD-67-positive GABAergic neurons in the hippocampus of saline-treated WT and GABA/CB1^−/−^ mice. *p < 0.05 using Mann-Whitney test; *n* = 36–39.

We also tested the possibility that the altered microglia activity in GABA/CB1^−/−^ mice was due to an altered expression of neuron-glia interaction proteins. Western blot analysis of hippocampal protein lysates (Figure 6A) revealed that the amount of the CX3CL1 was significantly reduced in the conditional knockouts (*t*_(16)_ = 2.399; *p* < 0.05; Figure 6B). The expression of the other neuronal interaction proteins CD200 (*t*_(10)_ = 1.403; *p* > 0.05), Sirpα (*t*_(10)_ = 1.013; *p* > 0.05) or their microglial receptors CD200R (*t*_(10)_ = 0.304; *p* > 0.05), CX3CR1 (*t*_(10)_ = 0.478; *p* > 0.05) and CD47 (*t*_(10)_ = 0.525; *p* > 0.05) did not differ between the genotypes (data not shown). Lastly, we asked how lack of CB1 receptors on GABAergic neurons influences the CX3CL1 expression on GABAergic neurons. For that, using confocal microscopy, we determined the intensity of CX3CL1 immunoreactivity in GAD67-positive cell bodies within the hippocampus. As shown in Figure 6C was clearly co-localized with GAD67 in WT mice. Importantly, GABAergic neurons in the hippocampus of GABA/CB1^−/−^ mice expressed significantly less CX3CL1 than GABAergic neurons in WT mice (Mann-Whitney *U* = 512; *p* < 0.05) and thus the co-localization was less evident (Figure 6D).

## Discussion

It was hypothesized that cannabinoid signaling plays an important role in the regulation of microglia cells (Luongo et al., 2010), because cannabinoid receptor agonist could decrease (Wolf et al., 2008) and antagonists increase (Lou et al., 2018) pro-inflammatory microglial activity. Our present study now suggests that cannabinoids can regulate microglial activity indirectly, through CB1 receptors expressed on GABAergic neurons, because genetic deletion of CB1 receptors from GABAergic neurons led to a pro-inflammatory microglial phenotype and altered reactivity to LPS in the hippocampus of GABA/CB1^−/−^ mice.

Generation and phenotypic analysis of constitutive (Ledent et al., 1999; Zimmer et al., 1999) and conditional CB1 receptor knockouts gave valuable information about the physiological role of cannabinoid receptors. The GABA/CB1^−/−^ mouse line was generated almost 15 years ago on a C57BL/6N genetic background (Monory et al., 2006). The conditional mutants are healthy, breed normally and have a relatively mild phenotype: They show hyperphagia (Bellocchio et al., 2010), recognition deficits (Albayram et al., 2016) and altered fear coping (Lafenêtre et al., 2009; Metna-Laurent et al., 2012). GABA/CB1^−/−^ mice have normal reactivity to acute stress (Steiner et al., 2008), acute THC-treatment (Monory et al., 2007) and to excitotoxic challenge (Chiarlone et al., 2014).

Microglia cells react to pro-inflammatory signals with morphological and functional changes. Activated microglia have an increased level of the ionic calcium binding adaptor molecule 1 (Iba1; Ito et al., 1998), enhanced cell body and they show an elevated expression of inflammatory cytokines like IL-6, IL-1β and TNF α (Hanisch and Kettenmann, 2007; Lynch, 2009).

In the absence of pro-inflammatory stimulus, microglia in the hippocampus of GABA/CB1^−/−^ mice had bigger cell bodies and increased TNFα level than in control WTs. The density of Iba1+ microglia was also higher in the hippocampus of saline-treated GABA/CB1^−/−^ mice, which may be the result of increased microglia numbers or due to an elevated Iba1 level. All these changes suggest an enhanced microglial pro-inflammatory activity in the hippocampus of GABA/CB1^−/−^ mice.

The up-regulation of the pro-inflammatory cytokine expression due to a massive pro-inflammatory stimulus by LPS injection was quite similar between the genotypes: the amplitude and dynamics of the cytokine expression was comparable between WT and GABA/CB1^−/−^ mice. The expression of the inflammatory cytokines was lower after repeated rather than after a single LPS injection in both genotypes suggesting a development of tolerance against the pro-inflammatory stimulus (Norden et al., 2016). However, the tolerance developed faster in the conditional knockout line, because 24 h after the fourth LPS injection the expression of each three cytokines remained elevated in WT mice, whereas in the conditional knockouts the TNFα expression did not differ between saline and LPS treated mice. It is important to note that the expression of cytokines was tested in the whole brain, whereas our histological analysis focused on the hippocampus. The inflammatory response to LPS can vary between brain regions, depending on the density of microglia (Pintado et al., 2011). On the other hand, microglia in the hippocampus showed similar morphological reactivity to LPS as in the prefrontal cortex, striatum or cerebellum (Verdonk et al., 2016).

Although microglia in GABA/CB1^−/−^ mice had a pro-inflammatory morphology, they showed a reduced morphological reactivity to LPS challenge. Microglia sizes increased 3 h after the injection in WT but not in GABA/CB1^−/−^ mice. 24 h after the LPS-treatment microglia in the GABA/CB1^−/−^ mice were significantly bigger than microglia in LPS-injected WT mice. Repeated LPS injection elicited a similar change in both genotypes. Microglia branching strongly differed between WT and GABA/CB1^−/−^ mice, as microglia branches in the hippocampus of knockout mice showed reduced size and complexity—typical for activated microglia. Interestingly, LPS-injection failed to induce any further change in branch morphology in the knockout mice. It is possible that the reason of the reduced microglial reactivity to LPS challenge is a ceiling effect. LPS could induce a significant reduction in microglial processes and concomitant increase in cell body size in WT mice, but not in the constitutive knockouts where microglia without LPS treatment already showed a pro-inflammatory morphology.

Furthermore, our results suggest that microglia morphology and cytokine expression are regulated independently from each other, in which is in accordance with previous publications (Cunningham et al., 2005; Norden et al., 2016).

Sex of the mice had a complex effect on LPS-induced increase in microglia activity assessed 24 h after a single injection: it did not influence cytokine production, whereas microglia density was higher in LPS-treated males than in females independent from the genotype. The increase in microglia size due to LPS treatment was generally more intensive in females than in males after LPS treatment in GABA/CB1^−/−^ mice. However, the sex of the mice did not influence the intensity of microglial size change in LPS-treated WT mice. When we compared the effect of LPS on microglia branching between the sexes we observed an opposite picture: reduction in branch size and complexity was more intensive in males rather than in females.

It is known that neurons control microglia activity by expressing surface proteins, which bind and activate their receptors expressed on microglia cells (Hoek et al., 2000; Cardona et al., 2006; Bessis et al., 2007). Besides these directly interacting neuron-glia ligand receptor pairs neurons detect glial activity by expressing cytokine receptors (Arisi, 2014; Crews and Vetreno, 2016) and microglia senses neuronal activity by expressing neurotransmitter receptors (Liu et al., 2016). Our study now extends the original hypothesis about the role of cannabinoid system in neuron–glia communication (Luongo et al., 2010) by showing the crucial role of GABAergic neurons in it. The cannabinoid signaling as a communication channel is probably most important between GABAergic neurons and microglia cells in the hippocampus because GABAergic neurons express cannabinoid receptors (CB1) on the highest level in this brain area (Marsicano and Lutz, 1999). The 2-AG production of microglia is high (Witting et al., 2004), while they express cannabinoid receptors at a very low level in the healthy brain (Stella, 2010). Deletion of CB1 receptors from GABAergic neurons disrupts the cannabinoid-mediated communication and through the lack of the necessary feedback from the microglia, GABAergic neurons could lose their inhibitory control. Indeed, reduced expression of CX3CL one is a sign of a diminished control and may be responsible for the pro-inflammatory phenotype of microglia in GABA/CB1^−/−^ mice (Cardona et al., 2006).

In a previous study, we found an increased density of activated microglia and enhanced expression of pro-inflammatory cytokines in 12-month-old GABA/CB1^−/−^ mice (Albayram et al., 2011). We had two alternative hypotheses for this phenomenon: We assumed that lack of CB1 receptors on GABAergic neurons exacerbates brain aging and, as a consequence leads to an increased microglia activity. Alternatively, it is possible that CB1 receptor signaling on GABAergic neurons has a specific role in the regulation of microglia activity, thus genetic deletion of CB1 from GABAergic neurons impairs neuronal control leading to accelerated brain aging. Our present results with young, 3-month-old mice support the second hypothesis and suggest that GABAergic neurons have a specific role in the regulation of microglia cells and cannabinoid signaling plays an important role in it.

## Author Contributions

FA, JK, EB, TZ, ÖA, DT and AB-G performed the experiments. ÖA, MH, AB-G and AZ designed and led the study.

## Conflict of Interest Statement

The author declares that the research was conducted in the absence of any commercial or financial relationships that could be construed as a potential conflict of interest.
